# The maximal gait speed is a simple and useful prognostic indicator for functional recovery after total hip arthroplasty

**DOI:** 10.1186/s12891-020-3093-z

**Published:** 2020-02-07

**Authors:** Manaka Shibuya, Yuta Nanri, Kentaro Kamiya, Kensuke Fukushima, Katsufumi Uchiyama, Naonobu Takahira, Masashi Takaso, Michinari Fukuda, Atsuhiko Matsunaga

**Affiliations:** 1 Department of Rehabilitation, Kitasato University Hospital, 1-15-1 Kitasato, Minami-ku, Sagamihara, Kanagawa 252-0375 Japan; 2 Department of Rehabilitation Sciences, Kitasato University Graduate School of Medical Sciences, 1-15-1 Kitasato, Minami-ku, Sagamihara, Kanagawa 252-0373 Japan; 3 Department of Rehabilitation, School of Allied Health Sciences, Kitasato University, 1-15-1 Kitasato, Minami-ku, Sagamihara, Kanagawa 252-0373 Japan; 4 Department of Orthopaedic Surgery, Kitasato University School of Medicine, 1-15-1 Kitasato, Minami-ku, Sagamihara, Kanagawa 252-0374 Japan

**Keywords:** Gait speed, Total hip arthroplasty, Functional recovery, Prognostic indicator

## Abstract

**Purpose:**

The present study aimed to compare the capabilities of preoperative usual and maximal gait speeds in predicting functional recovery in patients who have undergone total hip arthroplasty (THA).

**Methods:**

Primary and unilateral THAs were performed in 317 patients, and the proportion of patients who achieved unassisted walking (functional recovery) 5 days postoperatively was recorded as an outcome measure. Preoperative functional assessment included hip pain, leg muscle strength, range of motion (ROM), and gait speed evaluations. The capabilities of preoperative usual and maximal gait speeds in predicting functional recovery were compared based on the areas under the curves (AUCs) of receiver operating characteristic (ROC) curves. Further, ROC curves were constructed using two models: 1. a model of gait speed only and 2. a clinical model including age, sex, leg muscle strength, and ROM.

**Results:**

On the AUCs for predictive ability of functional recovery, maximal gait speed was greater than usual gait speed (0.66 and 0.70, respectively). The AUC for maximal gait speed was as large as that of the clinical model (0.70 and 0.70, respectively).

**Conclusion:**

Our results suggest that maximal gait speed is a simple and useful prognostic indicator of functional recovery in patients who have undergone THA.

## Introduction

Patients with end-stage osteoarthritis (OA) who undergo total hip arthroplasty (THA) demonstrate reduced pain and improved physical function and health-related quality of life [1]. Owing to rising healthcare-related costs and an increase in the demand for THA, it is necessary to develop a strategy for reducing the length of hospital stay and for the efficient and appropriate discharge of patients after treatment. Poor functional recovery during hospital stay is a common complication following major operations [2]. Preoperative identification of such patients using valid tools enables clinicians to plan postoperative resources accordingly [2, 3].

Conventional factors, such as age, sex, body-mass index (BMI), range of motion (ROM), and muscle strength can only limitedly justify the variance in the postoperative functional recovery among patients [4, 5]; in contrast, performance-based measures may predict functional recovery better than conventional patient factors [6]. In previous studies, it has been reported that functional indicators such as timed up-and-go (TUG) and sit-to-stand tests are useful in predicting postoperative recovery [7–11]. In addition, the Osteoarthritis Research Society International (OARSI) guidelines recommend a 30 s chair-stand test, a 40 m fast-paced walk test, and a stair-climb test as the core set of performance measures for OA patients [12]. However, due to restrictions such as advancing ages of patients and limited spaces for evaluation, all of these evaluations are often difficult to perform.

Evaluation of gait speed does not require a large space and can be performed in a short time even for elderly patients because it requires less time and less burden on the patient than conventional tests [13]. In addition, a recent systematic review reports that a detailed assessment of gait parameters before and after surgery can capture a patient’s functional recovery very clearly [14]. However, many of these studies predict a functional recovery time of two to three months after surgery. Although Oosting et al. [6] reported on the usefulness of usual gait speed for predicting functional recovery early in the postoperative period, the usefulness of maximal gait speed has not been investigated. As mentioned earlier, OARSI recommends assessment of maximal gait speed, and it is not clear whether the usual or maximal gait speed is useful for predicting early functional recovery. By comparing the predictive capabilities of preoperative usual and maximal gait speeds, the present study aimed to clarify the usefulness of gait speed in predicting functional recovery in patients who underwent THA.

## Methods

The study protocol was approved by the Ethics Committee of Kitasato University Hospital (permit number: B18–088), and was performed in accordance with the Declaration of Helsinki. Informed consent was obtained from all individual participants included in the study.

### Study population

This was a retrospective study. Primary and unilateral THA were performed in 336 patients at our hospital between November 2015 and March 2018. After excluding 11 patients who were unable to walk before surgery and 8 patients with perioperative complications including postoperative cerebral infarction (*n* = 1), load limitation due to bone vulnerability (*n* = 2), fractures during surgery (*n* = 2), and those with deep venous thrombosis that needed treatment early after surgery (*n* = 3), 317 patients were included in this study.

### Data collection

#### Clinical characteristics

Patient characteristics, including age, gender, BMI, comorbidities, preoperative functional assessment, length of hospital stay, and discharge, were collected from electronic medical records. In addition, time taken to achieve walking over a distance of 50 m with or without a walking aid and the proportion of patients achieving unassisted walking on postoperative day 5 were recorded as outcome measures. Functional recovery was evaluated daily and defined as the ability to walk over a distance of 50 m without human assistance, regardless of the use of a walking aid.

#### Preoperative functional assessment

Hip pain, knee extensor and hip abductor muscle strength, hip flexion ROM, and gait speed were assessed 1–3 days before operation by one of seven physical therapists who had received training for more than one month. Hip pain on the affected side during walking was assessed using a visual analog scale. Muscle strength was determined by measuring maximum voluntary isometric knee extensor and hip abductor strength on the affected side using a hand-held dynamometer with a restraining belt [15] (μTas; ANIMA, Tokyo, Japan). Measurements were obtained twice, with the highest values expressed as relative to body weight (%BW) used in the analysis. Hip flexion on the affected side was used to evaluate ROM. Usual and maximal gait speeds were measured by timing the patients walking at their usual or maximal pace with any necessary assistive devices over the middle 10 m of a 16-m walkway. A digital stopwatch was used to time subjects as they walked over a 10-m distance. Subjects were provided with 3 m to accelerate and decelerate before and after the test distance. For the usual speed walking trials, they were instructed to walk at their normal comfortable speed. For the maximal speed walking trials, they were asked to walk as fast as they could safely without running. First, usual gait speed was measured, and then, maximal speed was measured. Between each measurement, 30-s rest was taken.

### Standard management

A rehabilitation program is shown in Appendix. A standard rehabilitation program comprising weight bearing, as tolerated with a walking aid, was started on the day after surgery, and patients were allowed to eliminate walking aids whenever comfortable. Physical therapy was performed once a day on weekdays, and it included progressively improving walking ability, other functional activities, and walking stairs according to the needs and progress of an individual patient. Patients participated in a progressive program involving range of motion exercises, strengthening exercises, and functional training. Patients were allowed to use analgesics for pain as needed. Patients were discharged from the hospital on consultation with their caregivers, surgeons, nurses, and physical therapists, according to the following criteria: ability to walk independently with a walking aid, and if necessary, climb stairs, remain in a stable medical condition, and exhibit adequate wound healing. Discharge disposition in this study was defined as a rehabilitation facility or home, and the patients were transferred to a rehabilitation facility if they could not achieve the criteria at 1 week after surgery.

### Statistical analysis

Cox regression analysis constructing two predictive models was used to determine the prognostic capabilities of gait speed for predicting time to functional recovery: Model 1, age + sex; Model 2, Model 1 + hip and knee muscle strength + ROM.

We constructed receiver operating characteristic (ROC) curves to compare the predictive ability of usual and maximal gait speed for functional recovery on postoperative day 5. The areas under the curves (AUCs) of ROC curves were compared according to the method of DeLong et al. [16] We also constructed ROC curves for functional recovery at 5 days after the operation using two models: gait speed only and clinical model including age, sex, hip and knee muscle strength, and ROM.

The Kaplan–Meier method was used to evaluate functional recovery and comparisons were performed by the log-rank test. Patients were divided into 3 groups based on tertile of gait speed. Subgroup analyses of gait speed were performed to examine by means of Cox regression analyses with adjustment for age and sex as potential confounders, including age stratified at < 65 years, 65–74 years, and ≥ 75 years, and sex. Hazard ratios (HRs) are reported with corresponding 95% confidence intervals (CIs).

Data are presented as the means ± standard deviation or as percentages. Statistical analyses were performed using SPSS version 22.0 (IBM Corporation, Armonk, NY), STATA version 13.0 (StataCorp, College Station, TX), and R version 3.1.2 (The R Foundation for Statistical Computing, Vienna, Austria). In all analyses, *P* < 0.05 was taken to indicate statistical significance.

## Results

The characteristics of all patients included in the study are shown in Table 1. The patients had a mean age of 64.7 ± 11.8 years old, mean BMI of 24.5 ± 4.6 kg/m^2^, and 82.3% (261/317) were female. The mean time to functional recovery was 6.4 ± 3.1 days.

**Table 1:** Patient characteristics

Factor	*n* = 317
Age (years)	64.7 ± 11.8 (30–95)
Sex (*n*, %female)	261 (82.3%)
Body-mass index (kg/m^2^)	24.5 ± 4.6 (14.4–42.2)
Comorbidities, *n* (%)	
Lumbar disease	46 (14.5%)
Knee disease	19 (6.0%)
Diabetes mellitus	16 (5.0%)
Preoperative Functional Assessment	
Hip pain (affected side, mm)	56.4 ± 28.9 (0–100)
Knee extensor strength (affected side, %BW)	32.9 ± 13.6 (6.6–87.9)
Hip abductor strength (affected side, %BW)	18.2 ± 8.8 (2.3–47.2)
Hip flexion ROM (affected side, °)	87.8 ± 19.6 (20–132)
Usual gait speed (m/s)	0.89 ± 0.28 (0.22–1.56)
Maximal gait speed (m/s)	1.23 ± 0.42 (0.23–2.56)
Time to functional recovery (days)	6.4 ± 3.1 (2–23)
Length of hospital stay (days)	13.5 ± 4.5 (5–31)
Discharge to rehabilitation facilities, *n* (%)	71 (22.4%)

Values are presented as the mean ± SD (range), *n* (%)

%BW, percentage of body weight; ROM, range of motion

The associations of usual and maximal gait speeds with time to functional recovery as determined by Cox regression analyses are presented in Table 2. Even after adjusting for age, sex, muscle strength, and ROM (Model 2), the usual and maximal gait speeds were independently associated with functional recovery (HR = 2.32, 95% CI = 1.30–4.12; *P* = 0.004; HR = 1.97, 95% CI = 1.30–2.97; *P* = 0.001, respectively).

**Table 2:** Associations of gait speed with time to functional recovery

	Model 1			Model 2		
Variable	HR	95% CI	*P* value	HR	95% CI	*P* value
Usual gait speed	3.66	2.31–5.81	< 0.001	2.32	1.30–4.12	0.004
Maximal gait speed	2.57	1.90–3.48	< 0.001	1.97	1.30–2.97	0.001

Model 1: adjusted for age and sex. Model 2: Model 1 plus knee and hip muscle strength and ROM

*HR* hazard ratio, *CI* confidence interval

**Fig. 1:**
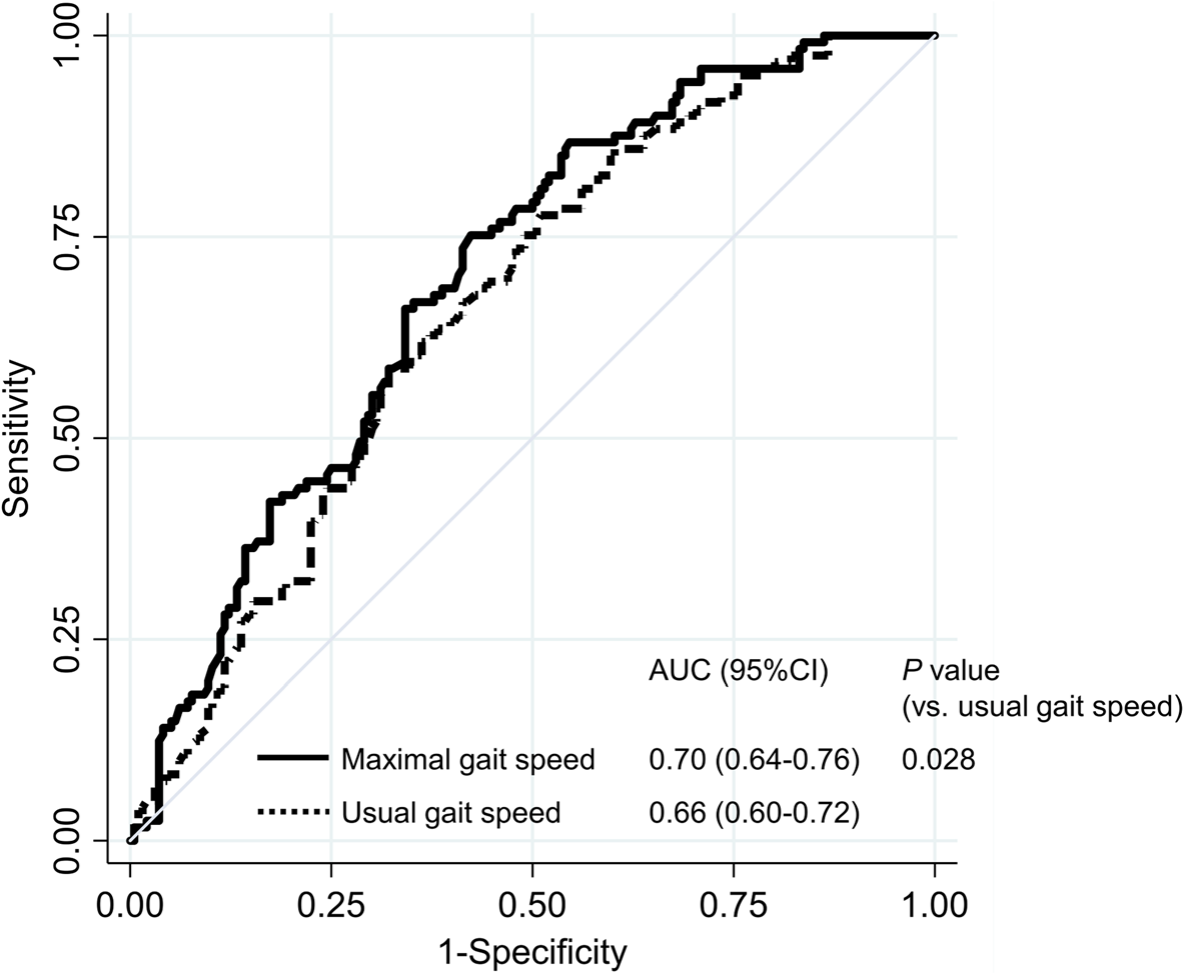
Receiver operating characteristic curves showing the predictive ability of usual and maximal gait speed for functional recovery. *Notes:* AUC, area under the curve; CI, confidence interval

**Fig. 2:**
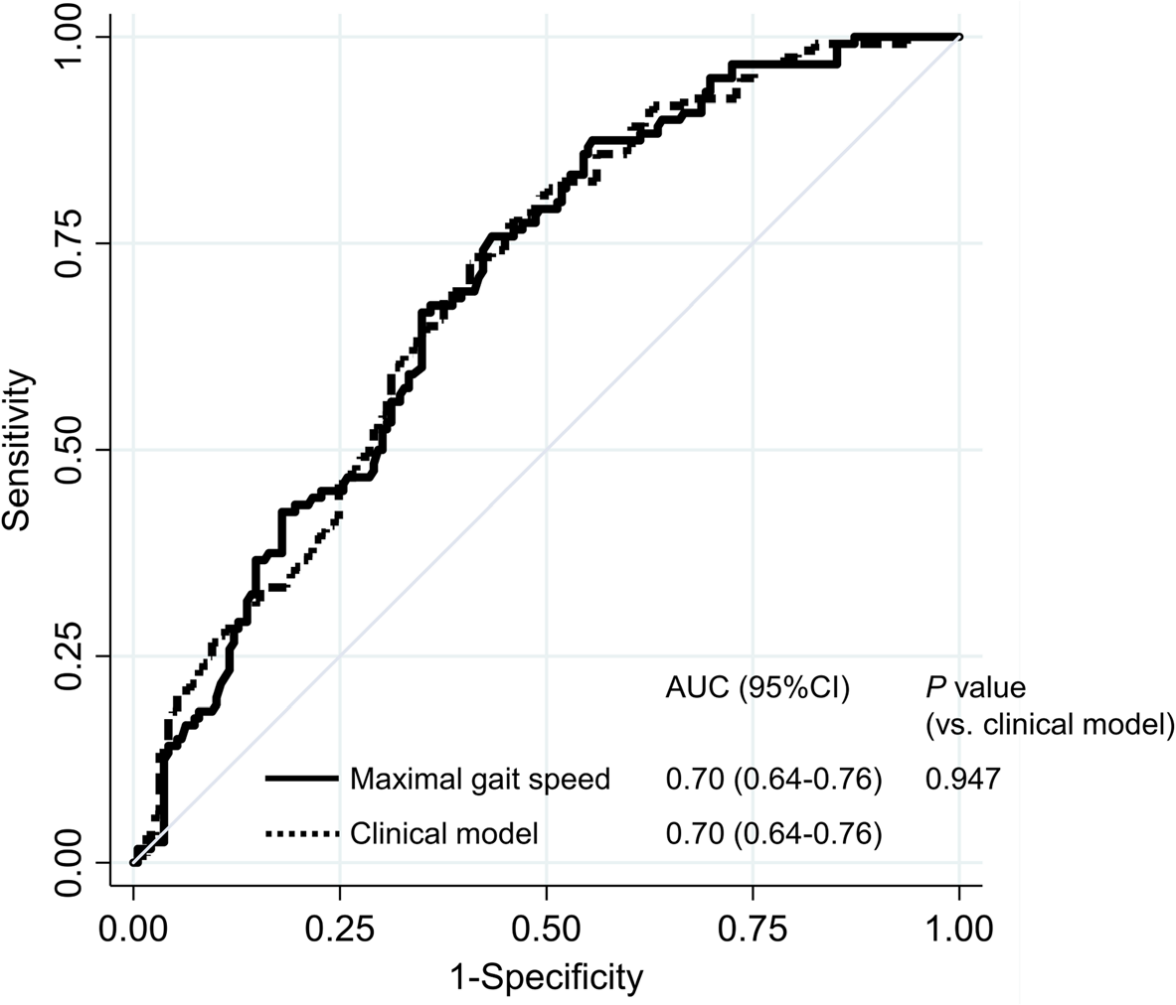
Receiver operating characteristic curves showing the predictive ability of gait speed and clinical model with regard to age, sex, muscle strength, and range of motion for functional recovery. *Notes:* AUC, area under the curve; CI, confidence interval

We compared the AUCs of usual and maximal gait speed using ROC analysis. Predictive ability of maximal gait speed for functional recovery on postoperative day 5 was significantly higher than those of usual gait speed (*P* = 0.028, Fig. 1). The AUC for maximal gait speed (0.70, 95% CI: 0.64–0.76) was as large as those for clinical model (0.70, 95% CI: 0.64–0.76) including age, sex, hip and knee muscle strength, and ROM as shown in Fig. 2 (*P* = 0.947).

**Fig. 3:**
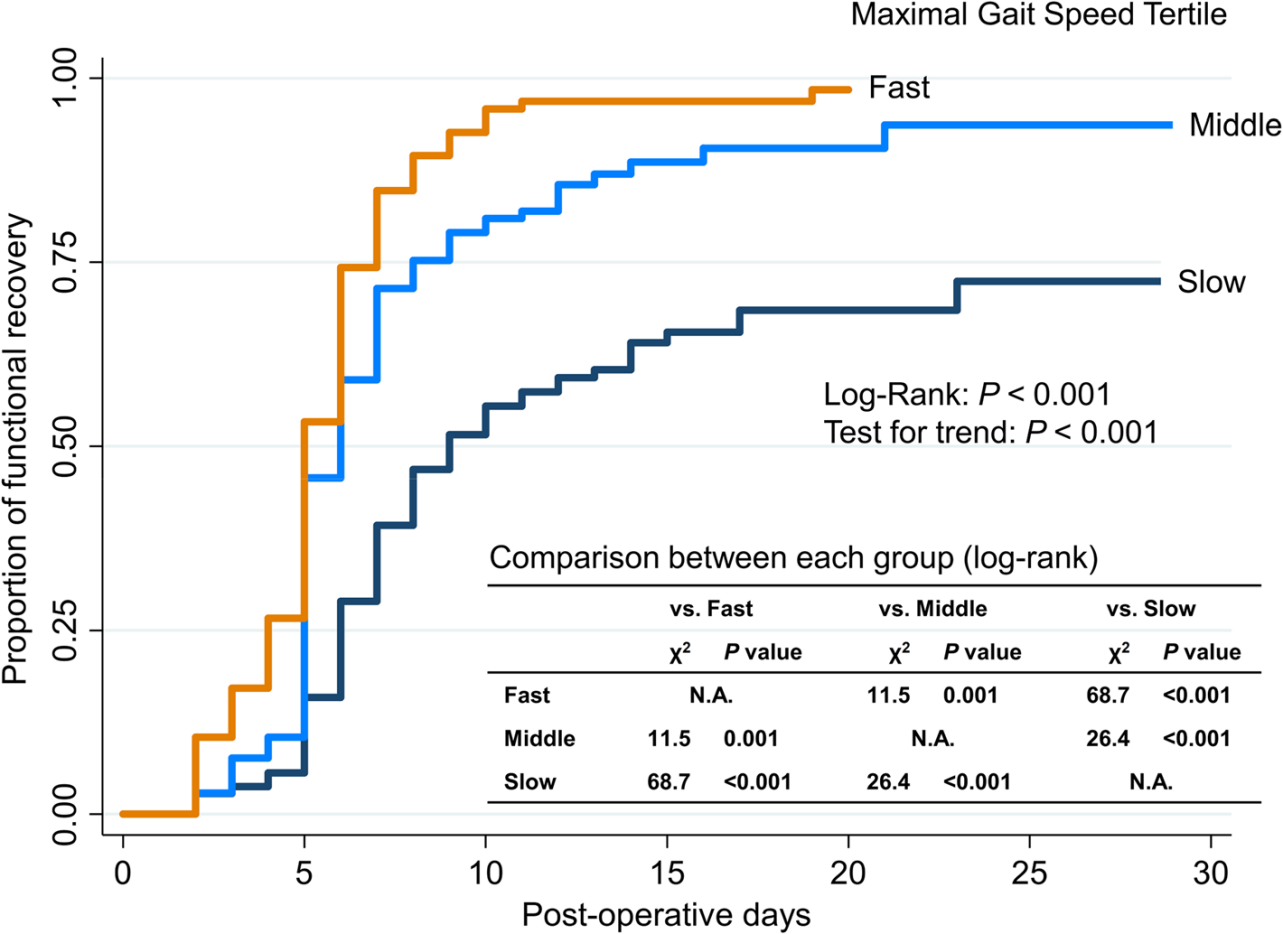
Kaplan–Meier curves for functional recovery according to tertiles of maximal gait speed. *Notes:* The fast, middle, and slow maximal gait speed tertiles were ≥ 1.41, 1.05–1.41, and ≤ 1.05 m/s, respectively

**Fig. 4:**
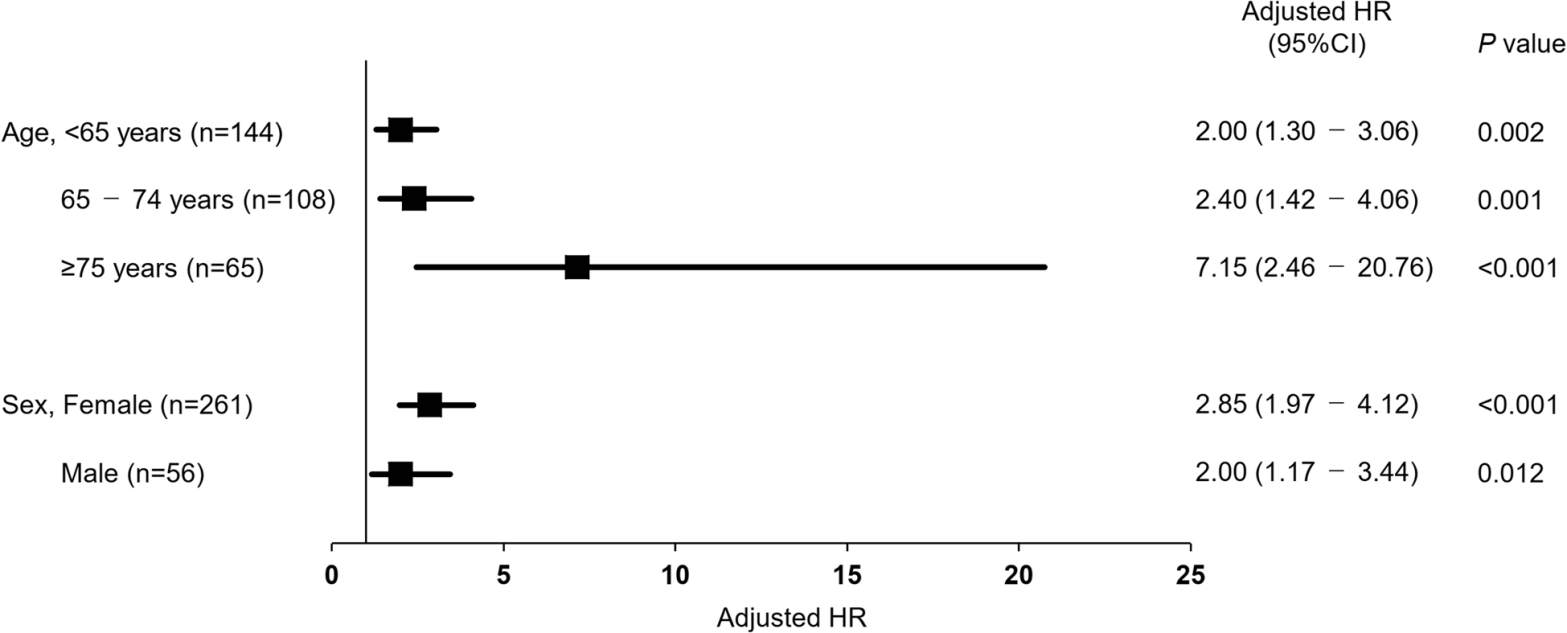
Forest plot showing the hazard ratios (HRs) for association of maximal gait speed with functional recovery. *Notes:* HR, hazard ratio; CI, confidence interval

On Kaplan–Meier analysis plotting tertiles of maximal gait speed, proportion of functional recovery was significantly higher in the fast gait speed group than the middle and slow gait speed group (log-rank, *P* < 0.001; Fig. 3). In addition, the middle gait speed group had also significantly higher proportion of functional recovery than the slow gait speed group (*P* < 0.001). Maximal gait speed was also consistently associated with the time to functional recovery across various subgroups after adjusting for age and sex as shown in Fig. 4.

## Discussion

The present study indicated an independent association between gait speed and functional recovery after THA. The predictive capability of maximal gait speed was higher than that of usual gait speed and equivalent to clinical model including age, sex, hip and knee muscle strength, and ROM. Patients with fast gait speed showed significantly faster functional recovery than those patients with slow gait speed. In addition, various subgroups of patients with fast gait speed consistently showed higher rates of functional recovery after adjusting for age and sex. Based on the results of the present study, maximal gait speed may be useful for predicting the functional recovery after THA.

Delay in functional recovery also contributes to prolongation of length of hospital stay. However, there have been few reports regarding factors related to functional recovery in inpatients following THA. Oosting et al. reported that age > 70 years old, presence of comorbidities, TUG score > 10.5 s, and usual walking speed < 1.0 m/s were risk factors for delayed functional recovery [6], while Elings et al. reported that male sex, age ≥ 70 years, BMI ≥25 kg/m^2^, American Society of Anesthesiologists score of 3, Charnley score of B or C, and TUG score of ≥12.5 s were significant risk factors [17]. In addition, Unnanuntana et al. reported that age, sex, and BMI were factors affecting ambulation distance at discharge as a measure of functional recovery [18]. These indicators appear to be important factors related to functional recovery. However, it is necessary to examine multiple factors for prediction, and this may be difficult from a practical viewpoint. The results of the present study indicated that maximal gait speed as a single index showed comparable predictive power as clinical model including age, sex, muscle strength, and ROM. The results of the present study suggested that preoperative assessment of maximal gait speed is a simple and useful predictive index for functional recovery after THA.

Previous studies have reported that functional indicators such as TUG and sit-to-stand test are useful in predicting postoperative recovery [7–10, 14, 19–22]. In addition, with the increasing age of patients who undergo artificial joint replacement, the importance of evaluating physical frailty has been reported in recent years [23]. Currently, various methods are used to assess physical frailty, but due to its simplicity and predictive capability, gait speed has become a core strategy to evaluate physical frailty. Gait speed has been shown to predict morbidity and mortality in various populations [24, 25]. In addition, the evaluation of gait speed is reliable and accurately measures even frail older adults with cognitive dysfunction, which is common comorbidity in the elderly population [26]. Importantly, several studies have indicated that the single measurement of gait speed outperformed other multicomponent frailty scales in predicting outcomes [27, 28]. Based on this available evidence, the European orthopedic expert group advises the use of gait speed to measure physical performance in daily practice [29]. These studies and guidelines also support the usefulness of gait speed, and the results of this study are consistent with these findings.

In recent reports of THA surgery, the average length of a hospital stay has been shortened year by year due to progress in surgery and the introduction of enhanced recovery after surgery (ERAS) [30]. Achieving walking independence in 5 days as reported in the current study is certainly a longer time than those reported above. According to a recent meta-analysis, the average hospital stay worldwide is 2–13 days, which varies greatly by country and medical facility, from early discharge being more common in the United States, to hospitalization of a week or longer usual for other countries [31]. In this study, functional recovery at 5 days was adopted with reference to a previous study [17].

## Limitations

This study had several limitations. First, this was a retrospective, single-center study. Second, although multivariate analysis was performed, many factors related to functional recovery have been reported, and other factors that were not measured, such as cognitive impairment, smoking history, and medication like analgesics [4, 32], may have resulted in residual bias. Third, the study population included only Asian patients after THA. Fourth, this study does not measure other performance tests such as TUG or sit-to-stand test, so it was not able to compare the usefulness of gait speed with other performance tests. Further studies are required to validate the utility of maximal gait speed as a predictor of functional recovery in other populations or as compared with other evaluations.

## Conclusion

Preoperative maximal gait speed was shown to be associated with early functional recovery following THA. The results presented here suggested that the preoperative maximal gait speed may be a simple and useful prognostic indicator for functional recovery after THA.

## Data Availability

The datasets generated and/or analysed during the current study are not publicly available due that individual privacy could be compromised, but are available from the corresponding author on reasonable request.

## Appendix

### Rehabilitation program

Day 1 after surgery:

- Cold pack or ice pack to manage pain, inflammation, and swelling
- Start exercises (ankle pumps and quadriceps sets)
- Start transfers
- Observe hip precautions

Day 2:

- Continue as above with emphasis on decreasing pain and swelling and promoting independence with functional activities
- Start walking with a standard walker for 50 m and weight bearing as tolerated
- Start passive/active assisted/active range of motion (P/AA/AROM) exercises
- Start muscle strengthening exercises (e.g., quadriceps sets in full knee extension and gluteal sets)
- Perform bed mobility and transfers with minimum assistance
- Observe hip precautions

Days 3–7:

- Continue as above with emphasis on achieving a proper gait pattern with an assistance device
- Perform bed mobility and transfers independently
- Ambulate with a T-cane for 50 m
- Start activities of daily living training (e.g., dressing activities, climbing stairs, cutting nails, and bathing activities)
- Instruct home exercises (e.g., supine: ankle pumps, quad sets, hamstring sets, gluteal sets, assisted heel slides, and hip abduction; seated: long arc quad and knee flexion; and standing: hip flexion with knee bend, knee flexion, heel raises, terminal knee extension, hip abduction, and mini-squats)
- Observe hip precautions

After Day 7:

- Continue as above
- Ambulate with a T-cane for 50 m independently
- If necessary, climb stairs independently
- Observe hip precautions

## Abbreviations

AUC: Area under the curve
BMI: Body-mass index
BW: Body weight
CI: Confidence interval
ERAS: Enhanced recovery after surgery
HR: Hazard ratio
OA: Osteoarthritis
OARSI: Osteoarthritis Research Society International
ROC: Receiver operating characteristic
ROM: Range of motion
THA: Total hip arthroplasty
TUG: Timed up-and-go
